# Avacopan as an add-on therapy in a paediatric patient with new-onset granulomatosis with polyangiitis and acute kidney injury: a case report

**DOI:** 10.1093/rheumatology/keaf523

**Published:** 2025-10-11

**Authors:** Robin Mørk, Pernille Bøyesen, Ingvild Andrea Kindem, Jacob Lilleby, Ragnar Gunnarsson, Øyvind Molberg, Vibke Lilleby

**Affiliations:** Department of Rheumatology, Oslo University Hospital, Oslo, Norway; Department of Rheumatology, Oslo University Hospital, Oslo, Norway; Department of Paediatric and Adolescent Medicine, Oslo University Hospital, Oslo, Norway; Faculty of Medicine, Riga Stradins University, Riga, Latvia; Department of Rheumatology, Oslo University Hospital, Oslo, Norway; Department of Rheumatology, Oslo University Hospital, Oslo, Norway; Institute of Clinical Medicine, University of Oslo, Oslo, Norway; Department of Rheumatology, Oslo University Hospital, Oslo, Norway

Rheumatology key message

- Avacopan appears to be a steroid-sparing, effective and safe add-on treatment for paediatric ANCA-associated vasculitis.

Dear Editor, Childhood-onset anti-neutrophilic cytoplasmatic antibody (ANCA)-associated vasculitis (AAV) is a rare disease with few paediatric data published. Despite advances in treatment, AAV remains a severe disease with frequent relapses and high cumulative morbidity. In a French study on childhood-onset AAV, 90% of the patients achieved remission after induction treatment, but after about 5 years of follow-up, 6% had died and 34% developed end-stage kidney disease [1]. Treatment-related adverse events, especially infections, are a major cause of mortality. The European vasculitis study (EUVAS) group reported a 1-year mortality rate of 11%, with 59% of deaths being therapy-related [2]. Highlighting the need for both effective and safe treatment alternatives.

The complement factor C5a plays an important role in the pathogenesis of AAV. Avacopan is an orally administered selective antagonist of the C5a receptor (C5aR) and has been shown to improve remission rates and kidney function in adults with AAV compared with oral glucocorticoids [3, 4]. There is limited experience with the use of avacopan in childhood-onset AAV. In our case, we initiated avacopan with the aim of preventing end-stage kidney injury.

We report the case of a 12-year-old girl diagnosed with granulomatosis with polyangiitis (GPA). She presented with a 2–4-weeks history of fever, sore throat, dry cough, recurrent epistaxis, nasal congestion and unilateral secretory otitis media. Initial antibiotic treatment for suspected pneumonia was ineffective. She was referred to our hospital following a rapid increase in serum creatinine from 134 to 350 µmol/l (ref.range 40–72) over four days and a markedly elevated PR3-ANCA IgG of >176 x10E3 IU/l (ref. <3). On admission, she exhibited haemoptysis. Blood tests showed anaemia (haemoglobin 8.4 g/dl; ref.range 11.7–15.3), elevated inflammatory markers (CRP 107 mg/l [ref. <4], ESR >100 mm/h [ref.range 1–10]) and severe kidney injury with creatinine 455 µmol/l, eGFR 12 ml/min/1.73m^2^, urea 20.3 mmol/l (ref.range 2.6–6.4), hypertension, proteinuria (total protein/creatinine ratio 105 mg/mmol, ref. range <30) and haematuria (10–30 erythrocytes/HPF), see Fig. 1.

**Figure 1.**
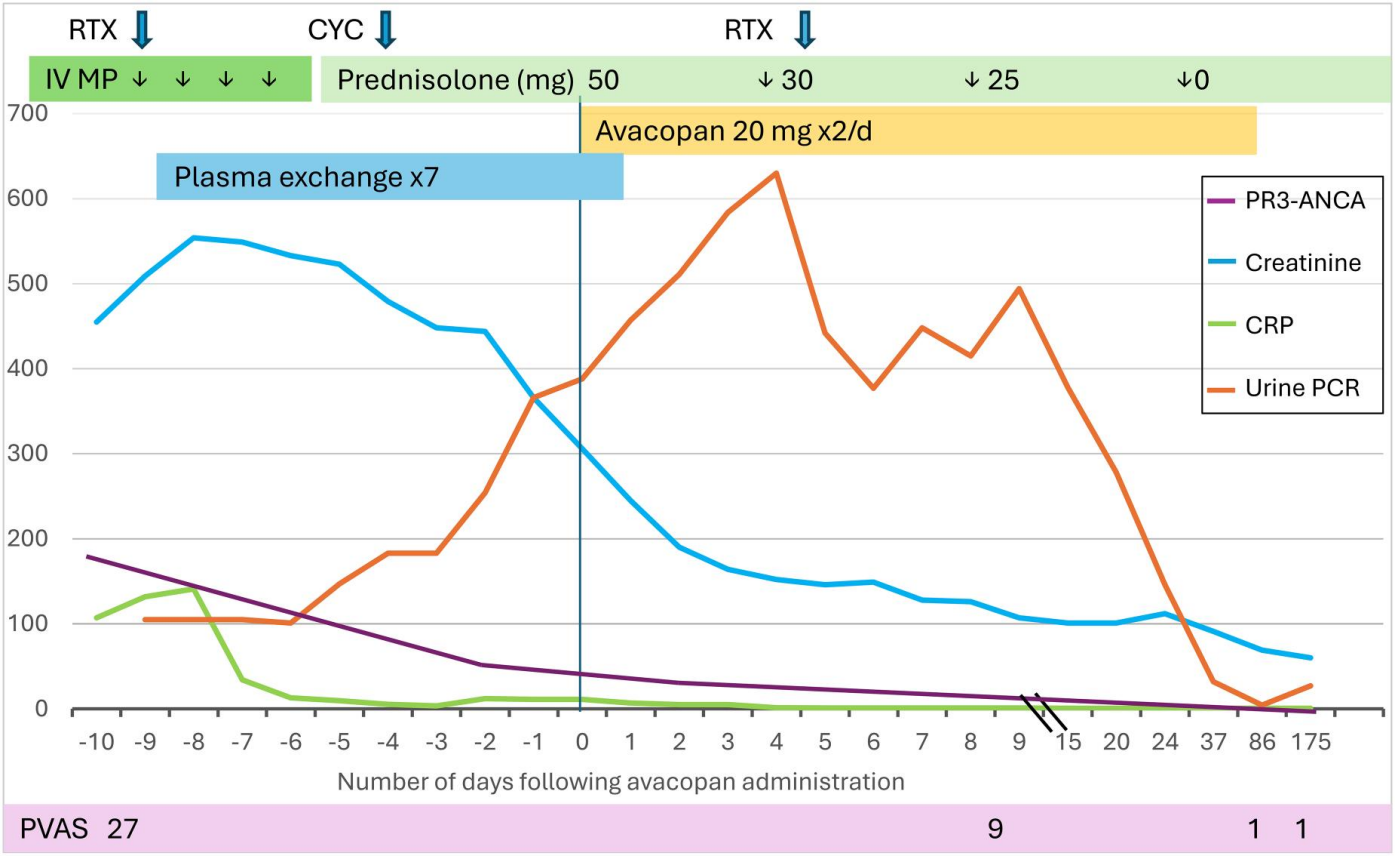
The clinical course of a 12-year-old patient treated with avacopan for granulomatosis with polyangiitis from admission at our centre to control 6 months later. The induction therapy with intravenous methylprednisolone (IV MP) 15 mg/m^2^, rituximab (RTX) 750 mg/m^2^, cyclophosphamide (CYC) 15 mg/kg and avacopan 20 mg BID is highlighted in the upper row. The *X*-axis shows the number of days following avacopan administration. The *Y*-axis shows the numerical value of each parameter colour-coded in the textbox; proteinase-3 anti-neutrophil cytoplasmatic antibody (PR3 ANCA) U/ml, creatinine µmol/L, CRP mg/L and urine PCR mg/mmol. The Paediatric Vasculitis Activity Score (PVAS) of the patient is indicated below the *X*-axis

Kidney biopsy revealed crescents in 23/24 glomeruli and acute tubulointerstitial inflammation. Chest CT showed multiple nodular lung consolidations and findings suggesting alveolar haemorrhage. Minor changes were found in her ethmoidal sinuses, mastoid cells and left middle ear. Otoscopy showed a ruptured tympanic membrane. The Paediatric vasculitis activity score (PVAS) was 27/63 points, reflecting high disease activity.

Induction therapy included intravenous methylprednisolone pulses, rituximab (RTX) and prednisolone. Due to rapid-progressive glomerulonephritis with creatinine reaching 509 µmol/l and eGFR 11 ml/min/1.73m^2^, plasma exchange was added with a total of 7 exchanges over 10 days (Fig. 1) [5]. Haemodialysis was required once due to oliguria and uraemia. A single cyclophosphamide (CYC) dose (15 mg/kg) was administered (day-4), both due to the severity of the disease and the potential RTX depletion that follows plasma exchange.

Due to rapid-progressive, severe kidney involvement, C5aR inhibition therapy with avacopan was approved for off-label use by the local health authorities. Avacopan was dosed 20 mg bid [4]. Kidney improvement was observed within two weeks after the start of induction therapy with decreasing creatinine (Fig. 1). Prednisolone was rapidly tapered from 50 mg qd (1 mg/kg) to discontinuation within 4 weeks (Fig. 1). By day 37 after introducing avacopan, PVAS improved to 9, CRP was normalised, and PR3-ANCA, creatinine and urine PCR were nearly normalised (Fig. 1).

At 3 and 6 months after initiating avacopan, she exhibited no active disease, with no proteinuria, creatinine at 60 µmol/l and eGFR at 94 ml/min/1.73m^2^. Hypertension was absent. PVAS improved further to 1, and the Paediatric Vasculitis Damage Index (PVDI) was 1 (ENT—hearing loss). Avacopan was well tolerated, with the only potential side effects being mild neutropenia and lymphopenia, which did not necessitate discontinuation of the treatment.

## Discussion

This case highlights avacopan’s potential as a steroid-sparing agent in childhood-onset GPA with severe kidney involvement. The rapid prednisolone taper and sustained remission suggest its efficacy and tolerability, though there are few published data on the use of avacopan in children.

To the best of our knowledge, this is the first report describing the successful use of avacopan as part of induction therapy in new-onset paediatric GPA, together with a description of the early clinical course and outcome. There are only three previous case reports published on avacopan use in paediatric patients [6–8]. All patients received 30 mg bid, and one developed severe liver function abnormalities which led to treatment discontinuation [7]. Results from an ongoing phase 3 trial on avacopan in childhood-onset AAV in combination with rituximab or cyclophosphamide are estimated in 2030 (NCT06321601).

While our patient demonstrated significant clinical improvement, the efficacy of the treatment could not solely be attributable to avacopan, given the concurrent administration of rituximab, plasma exchange and one course of cyclophosphamide. Nevertheless, avacopan facilitated rapid glucocorticoid (GC) tapering, consistent with its established steroid-sparing effect and recent incorporation in international consensus guidelines on adult-onset AAV [5].

GCs remain essential in AAV management for rapid disease control, but adverse events, including infections, cardiometabolic disease and therapy-related mortality, are well recognised. In recent years, there has been growing recognition of the benefits associated with the utilisation of lower induction doses or shorter courses of GCs in combination with CYC or RTX [5].

Avacopan add-on therapy offers the potential for substantially reduced GC exposure compared with the current standard of care. As observed in our patient, avacopan may serve as a steroid-sparing, effective and safe treatment option for paediatric AAV in the future. However, the access to both avacopan and rituximab might be limited globally, and alternative treatment strategies to reduce GC exposure are of clinical importance. Further research is warranted to determine optimal GC tapering strategies in conjunction with avacopan and other combination therapies, thereby ensuring patient safety and providing clear, evidence-based guidance for clinicians.

## Data Availability

The data underlying this article will be shared on reasonable request to the corresponding author.
